# Colon cancer exosome-derived biomimetic nanoplatform for curcumin-mediated sonodynamic therapy and calcium overload

**DOI:** 10.3389/fbioe.2022.1069676

**Published:** 2022-11-15

**Authors:** Yang Li, Chunyu Huang, Youhua Xu

**Affiliations:** ^1^ Faculty of Chinese Medicine, State Key Laboratory of Quality Research in Chinese Medicine, Macau University of Science and Technology, Taipa, Macao, China; ^2^ School of Pharmacy, State Key Laboratory of Quality Research in Chinese Medicine, Macau University of Science and Technology, Taipa, Macao, China; ^3^ Department of Gastrointestinal Surgery, Shenzhen People’s Hospital The Second Clinical Medical College, Jinan University, The First Affiliated Hospital, Southern University of Science and Technology, Shenzhen, Guangdong, China; ^4^ Department of Radiation and Medical Oncology, Hubei Key Laboratory of Tumor Biological Behaviors, Hubei Cancer Clinical Study Center, Zhongnan Hospital of Wuhan University, Wuhan, China

**Keywords:** exosome, curcumin, calcium carbonate, drug delivery, sonodynamic therapy

## Abstract

Sonodynamic therapy (SDT) possesses unique properties such as being minimally invasive, exhibiting low toxicity, as well as ability to impart the treatment in the deep tissues, and hence has been extensively used. However, inherent defects such as low water-soluble sonosensitizers can limit the clinical application of SDT, and tumor microenvironment (TME) can further compromise the effect of a single SDT. To overcome these challenges, we have designed a bionic nano-system (ECaC) by coating mesoporous calcium carbonate nanoparticles (CaCO_3_ NPs) and sonosensitizer curcumin (Cur) into tumor-derived exosomes for developing enhanced SDT. Exosome membrane could endow CaCO_3_ NPs with homologous targeting abilities. In addition, compared with the bare CaCO3 NPs, ECaC showed significant accumulation in the tumor cell species. Subsequently, CaCO_3_ NPs upon reaching the tumor site can be degraded into Ca^2+^ in response to the acidic microenvironment of the tumor to destroy the cellular mitochondria. Hence, the cellular respiration could be destroyed to be a vulnerable state, causing oxidative stress, enhancing Cur-mediated chemotherapy/SDT. This synergistically dynamic therapy has demonstrated significant anti-tumor effects under *in vitro* and *in vivo* settings without exhibiting any toxic side effects. Our prepared biomimetic nano-system can effectively deliver the hydrophobic Cur to the tumor sites, which holds great promise in field of drug delivery and can broaden the application of exosomes, as this method has a certain enlightenment effect on the subsequent development of exosomes.

## Introduction

According to the World Health Organization, cancer has become one of the major causes of mortality worldwide, and malignant tumors continue to be the leading cause of death (Zhu et al., 2020; Yu et al., 2021; Zhu et al., 2022a). The different traditional strategies used for tumor treatment display poor selectivity, toxic and side effects, and are generally can be only used in early-stage cancer patients (Baumann et al., 2016). Therefore, it is imperative to develop more effective and safe tumor treatment methods. In the past few decades, the applications of nanotechnology in the field of cancer treatment have developed rapidly (Chen et al., 2021a; Dai et al., 2021; Zhu et al., 2022b). Among them, non-invasive or minimally invasive treatment has become the preferred treatment method for researchers due to its advantages of precise positioning and relatively lesser side effects (Cui et al., 2021; Hu et al., 2021). It has been found that compared with the different traditional treatment methods, photodynamic therapy (PDT) can significantly reduce the toxicity to normal cells during the treatment process (Duo et al., 2021). However, due to the shallow penetration of light, the therapeutic effect of PDT on deeply located tumors located is rather limited. In addition, several photosensitizers display varying degrees of the phototoxicity, which makes them ineffective for the clinical application (Wang et al., 2021a). Sonodynamic therapy (SDT) is a non-invasive treatment method based on the interaction of ultrasound and sonoactive substances (sonosensitizers) (Zhang et al., 2021). In 1989, Yumita et al. first found that some organic substances could generate reactive oxygen radicals under the action of ultrasound, thus proposing the concept of SDT (Zhao et al., 2020). Although application of SDT has been associated with several advantages such as non-invasiveness, targeting, and little damage to normal human tissues, the clinical application effect of SDT is still unsatisfactory (Xu et al., 2020). Therefore, to further improve its therapeutic effect, nanotechnology, which has developed rapidly in recent years, can be utilized.

However, most sonosensitizers are aggregation-prone and weakly targeted, making their delivery very difficult (Liang et al., 2020). It has been established that the potential use of nanotechnology can improve the targeting of sonosensitizers, enhance their ROS yields or ultrasonic cavitation effects, and then promote the development of SDT in the biomedical field (Jiang et al., 2022; Wang et al., 2022). However, nanomaterials rely on the enhanced permeability and retention (EPR) effect to reach the tumor tissues but can be easily cleared by liver and kidney tissues as well as immune system. In recent years, enormous progress has been made in encapsulating nanomaterials with exosome membrane biomimetic technology to achieve specific targeting and avoid immune elimination (Guo et al., 2022; Ma et al., 2022). After the nanoparticles are encapsulated, the proteins on exosomes derived from the different cells are still biologically active, enabling them to escape from immune systems, prolong blood circulation, and directly target the tumor cells (Yang et al., 2021; Yuan et al., 2021). The exosome-encapsulated nanoparticles possess the properties of homologous targeting and homologous adhesion, which can enable them to specifically recognize and accumulate in the tumor tissues (Cao et al., 2019; Wang et al., 2021b). Huang et al. designed a novel tumor-derived biomimetic nanozyme to enhance radiotherapy (RT), and it was found that the tumor cell derived exosome membrane could endow FeS_2_ with optimal targeting ability and immune escape ability (Huang et al., 2021). Therefore, utilizing exosomes to deliver sonosensitizer can serve as a promising strategy to achieve SDT.

Calcium-based nanomaterials {such as calcium carbonate (CaCO_3_), calcium phosphate [Ca(H_2_PO_4_)_2_]} have good biocompatibility and biodegradability, and are widely used in chemical, medical and other fields (Bai et al., 2022). Calcium carbonate nanoparticles (CaCO_3_ NPs) remain stable at neutral pH and decompose into Ca^2+^ and CO_2_ at acidic pH, indicating their great potential for pH-responsive drug delivery and tumor therapy (Li et al., 2021). For example, Dong et al. developed monodisperse polyethylene glycol (PEG) modified CaCO_3_ nanoparticles as multifunctional nanocarriers for loading the photosensitizer [Ce6(Mn)] and chemotherapy drug doxorubicin (DOX) (Dong et al., 2016). The findings of *in vitro* experiments showed that CaCO_3_@Ce6(Mn)-PEG (DOX) nanoparticles remained stable at the physiological pH of 7.4, but could be rapidly degraded in a slightly acidic environment, and exhibited significant anti-tumor effects during treatment upon exposure to the combination of photodynamic therapy and chemotherapy. Similarly, Chang and co-workers designed a core–shell Cu_2_O@CaCO_3_ nanostructure for the synergistic oncotherapy (Chang et al., 2020). It was observed that Cu_2_O@CaCO_3_ could activate an immune-favorable TME for profound immune responses to simultaneously inhibit tumor distant metastasis and recurrence. Furthermore, Ca^2+^ can effectively destroy the mitochondria, leaving the cells in a vulnerable state, thereby sensitizing the effectiveness of SDT (Tan et al., 2021). In addition, cellular respiration was inhibited and oxygen content increased after the mitochondrial destruction. These properties can make calcium carbonate as a promising compound to cooperate with SDT to achieve tumor therapy.

**SCHEME 1:**
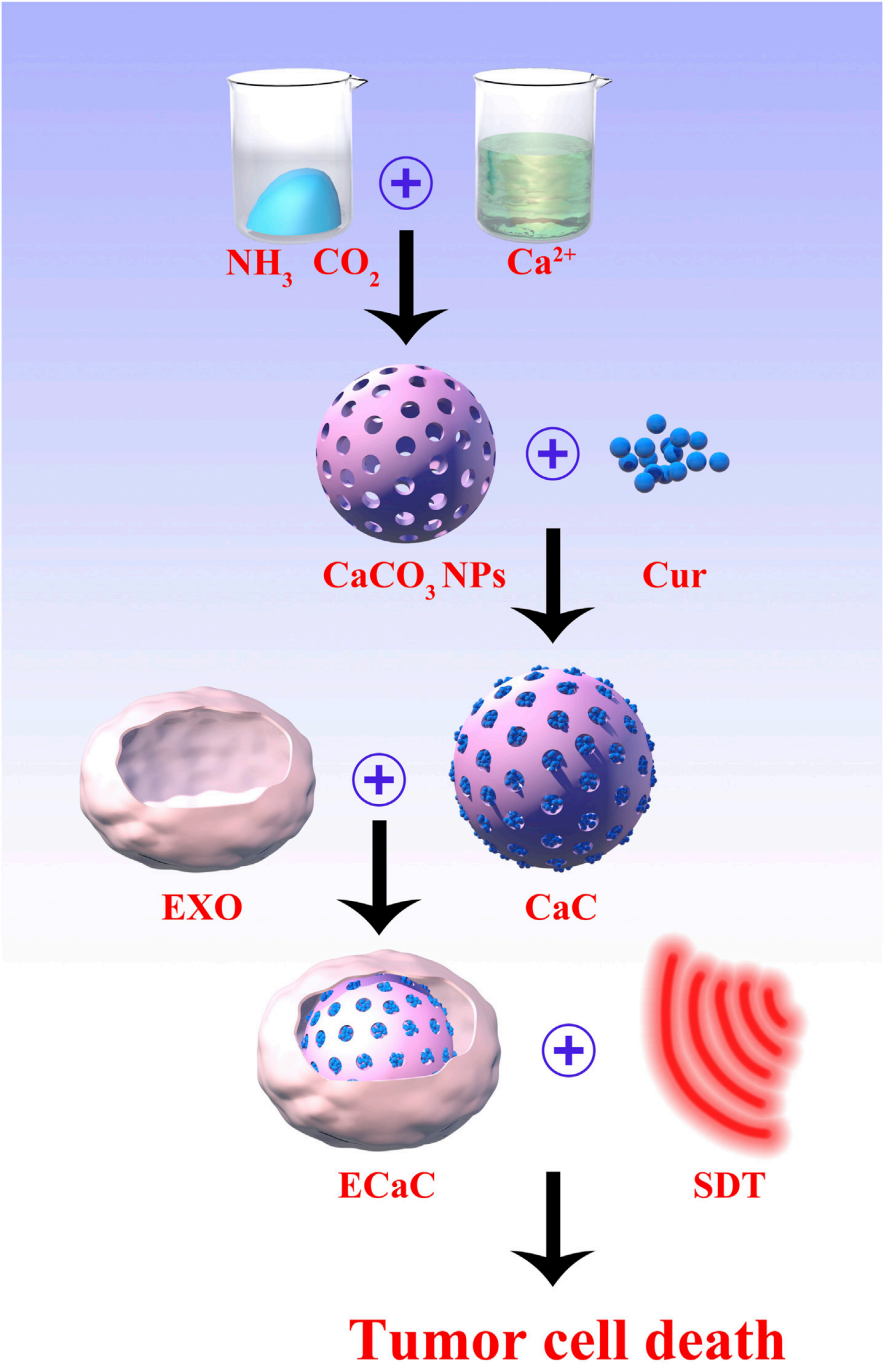
The scheme of colon cancer exosome-derived biomimetic nanoplatform for curcumin-mediated sonodynamic therapy and calcium overload.

In this study, by coating porous CaCO_3_ NPs and Cur with the tumor cell derived exosomes, a composite ECaC nano-system was generated (Scheme 1). The exosome membrane can endow CaCO_3_ with a new immune evasion ability, which can enable it to actively evade the clearance of organs such as liver and kidney, and specifically targets the tumor site, to facilitate the release Cur and Ca^2+^ in response to the degradation of the tumor’s acidic microenvironment. Ca^2+^ can effectively destroy the mitochondria of the tumor cells, thus rendering the cells in a state of inactivation, which is more conducive to the subsequent SDT activated by Cur. Cur also can act as a Ca^2+^ regulator, inhibiting Ca^2+^ efflux by stimulating Ca^2+^ release from the endoplasmic reticulum to the cytoplasm (Zheng et al., 2021). This treatment method can not only achieve the purpose of precise targeting, but also could significantly prolong the metabolic time of Cur and improve the treatment efficiency. Both *in vitro* and *in vivo* experiments have verified that ECaC combined with United States can achieve an optimal tumor treatment effect with good biological safety. This ECaC nano-system possesses useful clinical potential, and our experimental results can further expand the application of exosome-based nano-delivery systems.

## Results and discussion

**FIGURE 1:**
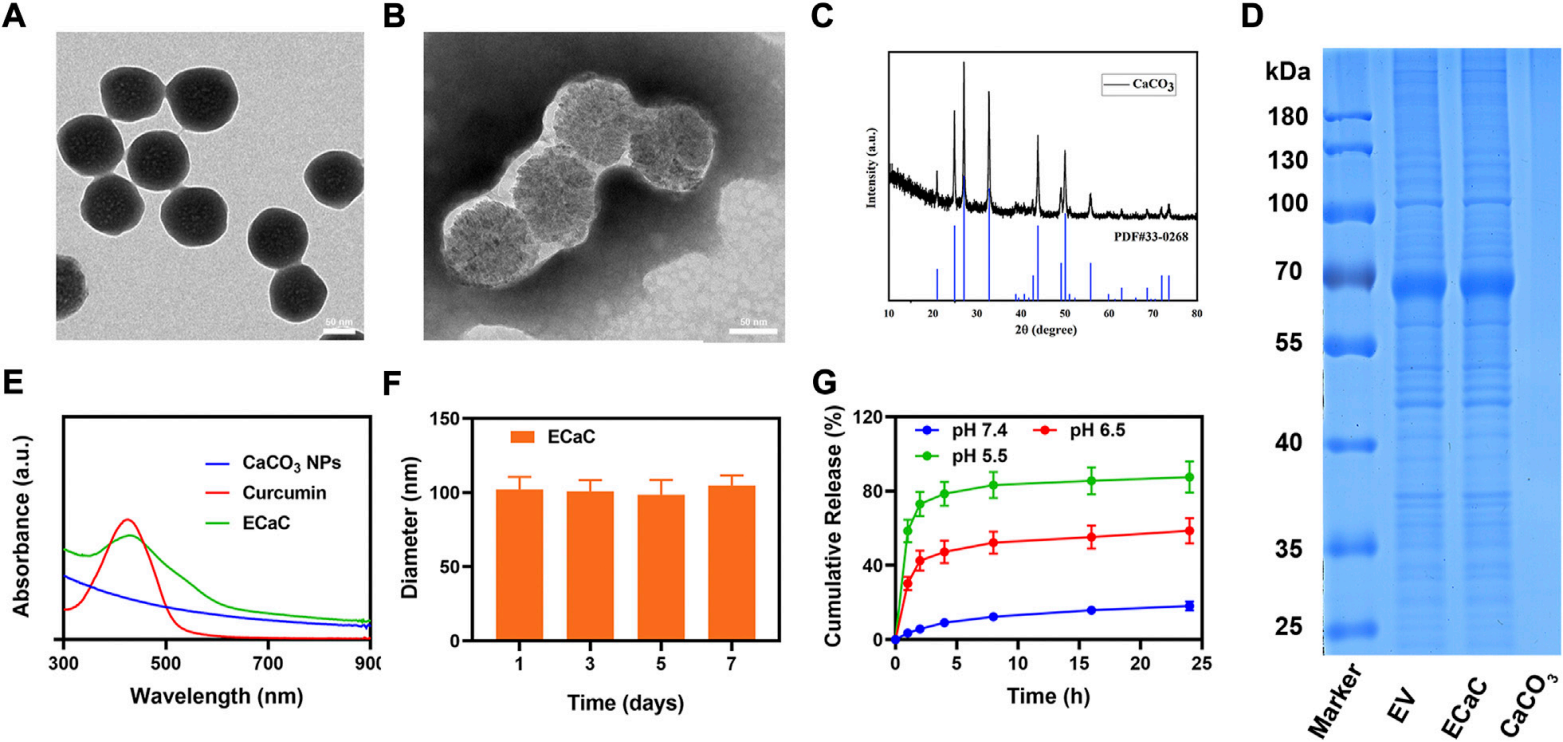
**(A)** TEM image of mesoporous CaCO3 NPs. **(B)** TEM image of ECaC. **(C)** X-ray diffraction (XRD) analysis of CaCO3 NPs. **(D)** Coomassie-stained SDS-PA gel image showing the protein composition of EV, ECaC and CaCO3. **(E)** Absorbance spectrum of CaCO3 NP, Curcumin and ECaC. **(F)** Statistical graph of the measured diameter of ECaC. **(G)** The release of Ca2+ from ECaC after incubation in PBS with different pH values (5.5, 6.5 and 7.4) for several hours.

Firstly, porous calcium carbonate nanomaterials (CaCO_3_ NPs) with uniform size were prepared by using a simple gas diffusion method. The results of transmission electron microscopy (TEM) indicated that the size of CaCO_3_ NPs was about 92 nm (Figure 1A). Thereafter, after short-term incubation with CT26 cells, ECa was isolated and purified using an exosome kit. Under the similar conditions, Cur was added for co-incubation and purification to obtain ECaC. As shown in Figure 1B, there was a thin exosome membrane formed on the surface of ECaC. X-ray diffraction (XRD) analysis (Figure 1C) showed that the prepared CaCO_3_ was consistent with the standard card (JCPDS No. 33–0268). X-ray spectroscopy (EDX) (Supplementary Figure S1) result shows that CaCO_3_ NPs contained three elements namely Ca, O and C. To further verify the successful modification of exosome membranes, we subjected exosomes, ECaC and pure CaCO_3_ to SDS-PAGE analysis, and the results indicated that there was no protein on the pure material, whereas ECaC and EV exhibited the same proteome (Figure 1D). The exosomes extracted from the tumor cells can successfully target the tumor cells and can be effectively taken up by the parental cells. This can enable rapid release of the CaCO_3_ and Cur loaded in them to exhibit the significant anti-tumor effects. Figure 1E showed the ultraviolet-visible absorption of various materials, and the results indicated that ECaC retained the characteristic absorption peak of Cur at about 427 nm, indicating that Cur was successfully loaded into ECaC. Moreover, the modification of the exosome membrane can aid to markedly improve the stability of the nanomaterials, as shown in Figure 1F, the particle size of ECaC was unchanged for 7 consecutive days, suggesting its potential for future biomedical applications, which is very important. Although many nanomaterials can exhibit substantial antitumor abilities, their instability can inhibit their future development for clinical use (Zhou et al., 2021). We continued to validate the acid-responsive degradation characteristics of CaCO_3_, and designed different pH environments to explore the possible release effect of Ca^2+^ in ECaC. As shown in the Figure 1G, CaCO3 released nearly 80% of Ca^2+^ in less than 12 h at pH 5.5. In conclusion, ECaC can actively target the various tumor tissues and can be specifically released in response to the specific microenvironmental properties of the tumors.

**FIGURE 2:**
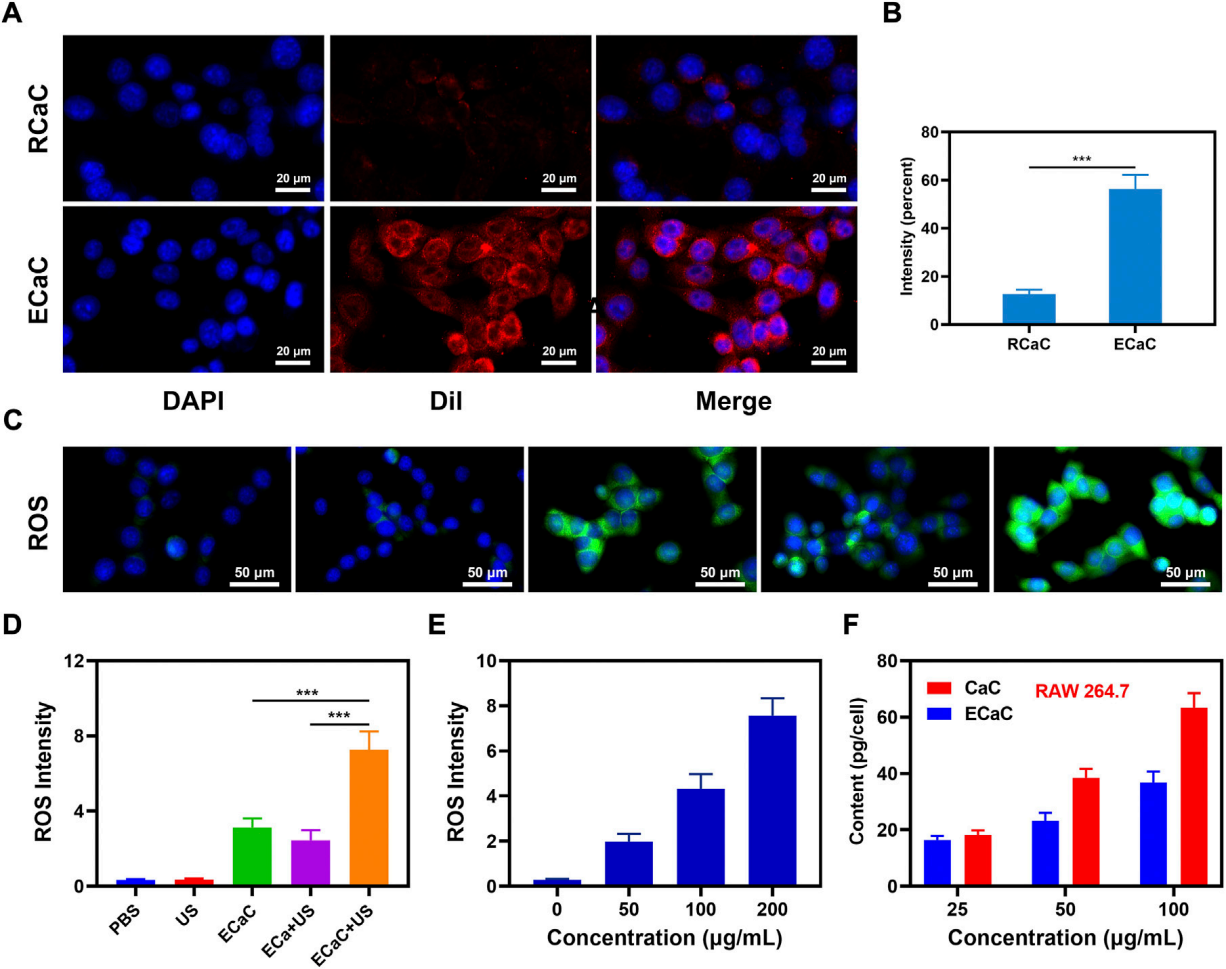
**(A)** Co-localization of DAPI (blue) and Dil (red) for RCaC and ECaC over time in CT26 tumor cells. **(B)** Dil fluorescence intensity of A determined using ImageJ software. **(C)** DCFH-DA fluorescence following the indicated treatments. **(D)** ROS fluorescence intensity of C determined using ImageJ software. **(E)** ROS fluorescence intensity of different ECaC concentration under United States treatment. **(F)** Nanoparticle uptake by RAW 264.7 cells at different incubated concentration. ***p < 0.001; Student *t*-test.

In view of the positive results of material characterization experiments, we continued to explore its potential further under *in vitro* settings, and prepared erythrocyte membrane-coated CaC and Cur by a similar method, named RCaC. The ECaC and RCaC were labeled with Dil and subjected to *in vitro* endocytosis experiments. The results showed that ECaC exhibited good targeting, and although RCaC also retained the proteins on the erythrocyte membrane, but it did not display any targeting potential (Figures 2A,B). Supplementary Figure S2 also illustrates the targeting potential of ECaC. In addition, intracellular reactive oxide species (ROS) production was detected using 2′, 7′-dichlorodihydrofluorescein diacetate (DCFH-DA), which could be oxidized to 2′, 7′-dichlorofluorescein (DCF) with green fluorescence in the presence of ROS. As shown in Figures 2C,D, the PBS and United States groups exhibited relatively weaker green fluorescence, which was primarily caused by the abnormal growth of tumor cells. There was only a slight fluorescence intensity in the ECa + United States group, which was caused by the destruction of the cell mitochondria after Ca^2+^ accumulated into the tumor cells. It is worth noting that ECaC can exhibit a moderate fluorescence effect, ECaC combined with United States can lead to maximal ROS production that can be attributed to the property of chemotherapeutic drug Cur to promote oxidative stress and inhibit the efflux of Ca^2+^, which can further promote the destruction of mitochondria by Ca^2+^. A significant ROS generation effect was achieved (Figure 2E), and Cur acted not only as a chemotherapeutic drug to amplify oxidative stress, but also serve as a sonosensitizer to achieve excellent SDT effect to enhance ROS content. The immune system can actively recognize and remove the foreign invaders. For example, the macrophages can remove the nanomaterials and pathogens in the form of fixed cells or free cells. We used RAW264.7 cells for entosis experiment, and found that pure CaC was significantly phagocytic, whereas the relative phagocytic amount of ECaC was significantly reduced, indicating that ECaC possessed good immune evasion characteristics (Figure 2F).

**FIGURE 3:**
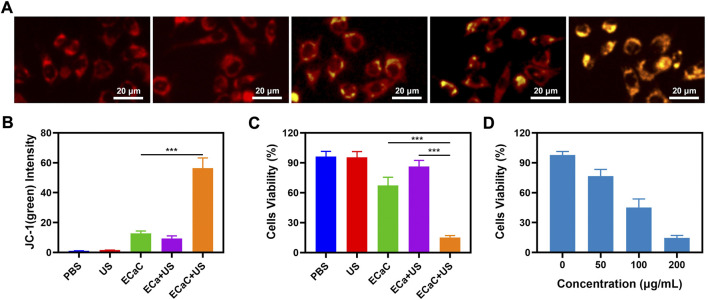
**(A)** JC-1 (green) for JC-1 monomer and red for JC-1 aggregate fluorescence image under different treatment. **(B)** Relative JC-1 (green) intensity in 3A. **(C)** The survival of CT26 cells with Control, United States, ECaC, United States + ECa and United States + ECaC detected by MTT assay. **(D)** The survival of CT26 cells with different ECaC concentration under United States detected by MTT assay. ***p < 0.001; Student *t*-test.

The change of the cellular calcium can aid to maintain a dynamic balance due to the combined action of the plasma and cellular mitochondria. When the intracellular calcium content is substantially higher than the normal level, the structure of mitochondria can be damaged, which in turn can compromise the function of mitochondria (Chen et al., 2021b). Hence, the changes in the mitochondrial membrane potential (MMP) in CT26 tumor cells were monitored by using JC-1 (5,5′,6,6′-tetrachloro-1,1′,3,3′-tetraethyl-imidacarbocyanine) probe method. JC dyes can typically accumulate in mitochondria, where they can clump together and fluoresce red. However, when mitochondria are damaged and MMP levels are low, JC monomers are released into the cytoplasm, producing green fluorescence (Zhu et al., 2022c). As shown in Figures 3A,B, PBS and United States groups exhibited red fluorescence. The merged mitochondria fluorescence of ECaC + United States group indicated maximum mitochondrial damage. Thereafter, we used MTT kit to verify the cell viability under the different treatment conditions (Figure 3C). The ECa combined with United States group only exhibited a slight therapeutic effect, but ECaC displayed certain tumor cell killing ability. The Ca^2+^ regulator Cur could stimulate the release of Ca^2+^ from the endoplasmic reticulum to the cytoplasm and can inhibit the outflow of Ca^2+^ from the cytoplasm to the extracellular fluid, enhancing the killing effect of Ca^2+^ on cells. Moreover, Cur itself also exhibited significant chemotherapeutic effects. Similarly, cell viability decreased with increasing ECaC concentration under United States irradiation (Figure 3D). ECaC + United States exhibited the best therapeutic effect, which was primarily caused by Ca overload and Cur-mediated SDT synergy.

**FIGURE 4:**
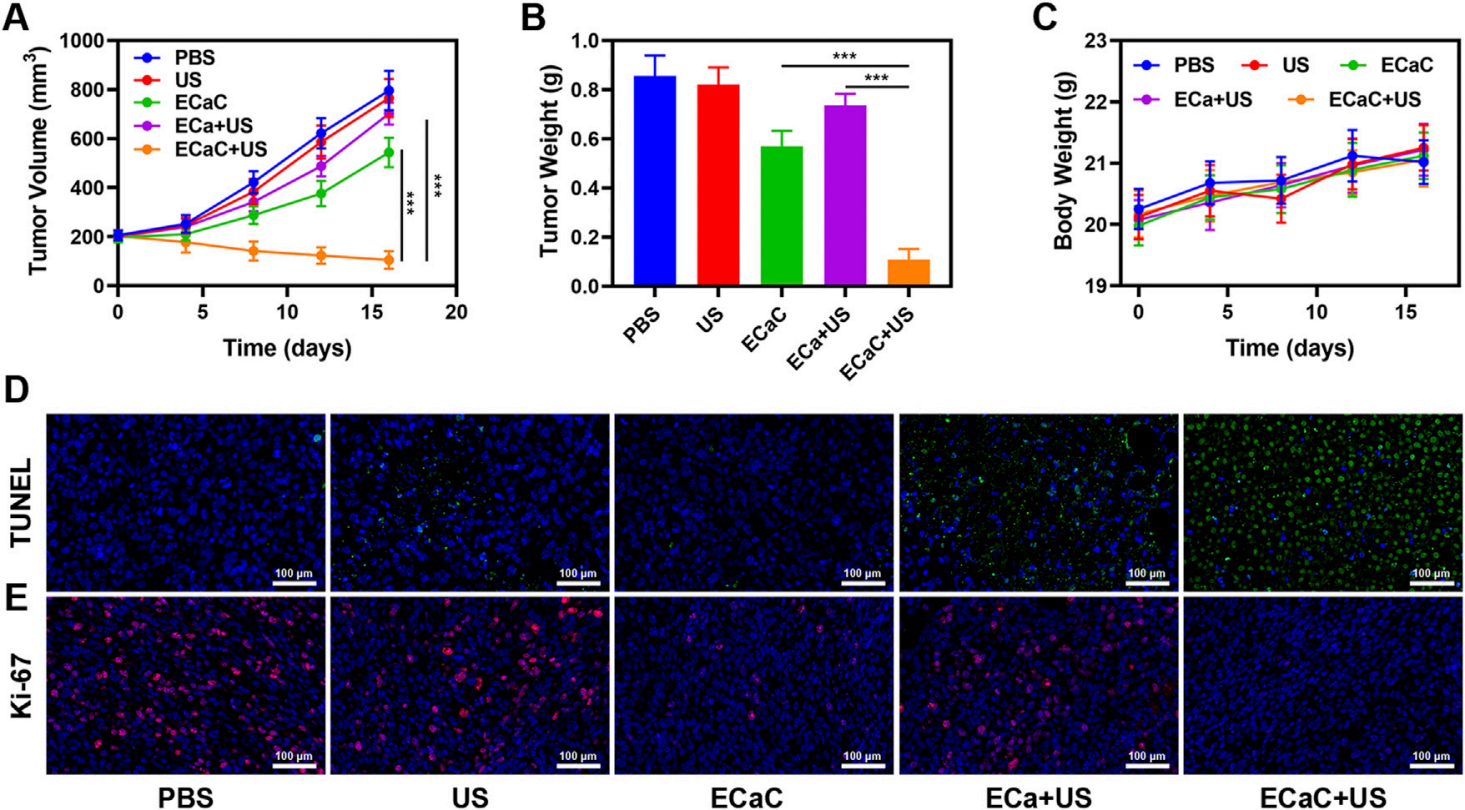
**(A)** Tumor growth curves of different groups of mice after various treatments. **(B)** Average weights of tumors collected from mice at day 16 after various treatments indicated. **(C)** The body weight variation of CT26 tumor-bearing mice during treatment. **(D)** Histological analysis of tumor after various treatments indicated. TUNEL (upper) and **(E)** Ki-67 (bottom) stained slices of tumors were collected from mice one day after various treatments indicated. ***p < 0.001; Student *t*-test.

**FIGURE 5:**
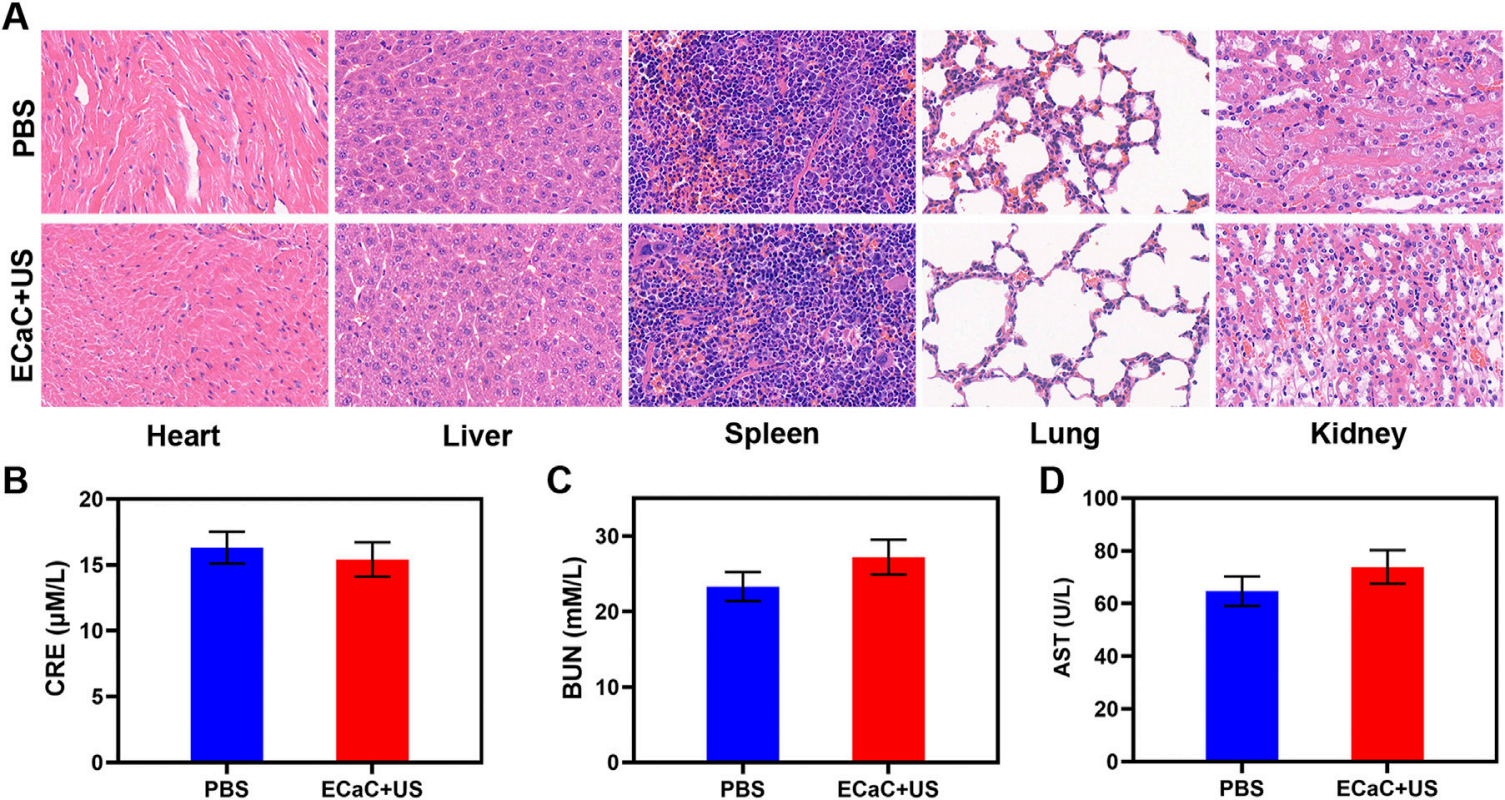
**(A)** Histopathological analysis results (H&E stained images) of the major organs, heart, lung, liver, kidneys, and spleen, of mice that were exposed to different treatments 16 days post-injection. Scale bars: 100 μm. (**B–D**) Liver, kidney and blood function markers: AST, CRE and BUN after various treatments.

The safety of nano-formulations injected into organisms cannot be completely ignored, and the targeted delivery of ECaC to the tumor cells is achieved through the enhanced permeability and retention effect of exosome membrane coating and nanomaterials. Exosome membrane modification can enable ECaC to possess immune evasion ability during blood circulation, thereby avoiding the massive enrichment of ECaC in the normal organs, and thereby avoiding long-term systemic toxicity and toxic side effects. We conducted *in vivo* pharmacokinetic experiments to study the potential effect of exosome membranes on blood retention. As shown in Supplementary Figures S3, S4, both ECaC and RCaC had long cycling properties, while CaC could guarantee a longer Ca retention time. Moreover, *in vivo* organ biodistribution profile showed that a large amount of ECaC reached the tumor site through active targeting 12 h after injection, and exhibited a lower dose accumulation in the liver and kidney than CaC. Based on the above results, we continued to explore ECaC-based *in vivo* anti-tumor effects. In this study, CT26 cells were injected subcutaneously into the mice to establish a CT26 colon cancer subcutaneous tumor model. Once the tumors reached approximately 200 mm^3^ in size, tumor-bearing BALB/c mice were randomly assigned into five different groups: 1) PBS; 2) United States; 3) ECaC; 4) ECa + United States and 5) ECaC + US. Each group was given corresponding treatment. Groups 3, 4 and 5 were administered a 10 mg/kg dose of CaCO3 NPs. After 12 h of intravenous injection, United States irradiation (power density = 1.5 W/cm^2^, transducer frequency = 1 MHz, 30% duty cycle, 10 min) was carried out. The tumor volume was measured with the digital calipers after every 4 days and the tumor weight was finally calculated. The results of the study indicated that the tumor volume increased rapidly in the control group, but the tumor growth curve of ECa without Cur combined with United States was only slightly suppressed. In the ECaC group containing the chemotherapeutic drugs Cur and CaCO_3_ NPs, the initial tumor volume growth in this group exhibited a certain retention effect, but then grew rapidly. The ECaC synergistic United States group showed the maximal tumor suppressor effect, suggesting that the two treatments have a stronger synergistic antitumor effect (Figures 4A,B). In addition, during the treatment period, the weight of the mice in each group increased steadily, and there was no significant change (Figure 4C), indicating that the ECaC system could not cause acute damage to the body. After the treatment cycle, the tumors in the different groups were also harvested and photographed. Deoxynucleotidyl transferase-mediated dUTP nick end labeling (TUNEL) and Ki-67 staining of the tumor slices demonstrated that ECaC combined with United States group exhibited the highest level of tumor cell apoptosis with the lowest level of tumor cell proliferation (Figures 4D,E). After the treatment cycle, mice in all the four groups were euthanized, followed by collection of the major organs for further analysis and blood for biochemical analysis (Figure 5; Supplementary Figures S5, S6). The relevant results of both the experimental group and the conventional control group showed that the mice functioned normally after the treatment, and our treatment method did not show any short-term adverse effects.

## Conclusion

In conclusion, a novel nano-biomimetic system for the simultaneous delivery of CaCO3 NPs and the chemotherapeutic drug Cur was designed to achieve robust SDT. ECaCs with exosome membrane modifications could be specifically targeted to the tumor sites, evade clearance by the immune system, and accumulate more effectively at the tumor sites. Subsequently, it can be gradually degraded in the weak acid environment of the tumor, and the Ca^2+^ and Cur are released. After Ca^2+^ causes damage to the mitochondria, the cells are more easily impaired and the Cur-mediated SDT was markedly strengthened. The tumor volume did not increase significantly during the treatment and the main organ parameters of the mice were found to be normal after the treatment, indicating the potential safety of the ECaC system. Overall, ECaCs synthesized by us highlight the promising application of exosomes for drug delivery.

## Data Availability

The original contributions presented in the study are included in the article/Supplementary Material, further inquiries can be directed to the corresponding author.
