# Supplementary material for: Colon cancer exosome-derived biomimetic nanoplatform for curcumin-mediated sonodynamic therapy and calcium overload
Source: Front Bioeng Biotechnol. 2022 Nov 15;10:1069676. doi: 10.3389/fbioe.2022.1069676 (PMC9705788; doi:10.3389/fbioe.2022.1069676)
Supplement: Supplementary file 1 [file Table1.DOCX]

**Materials**

The process of obtaining deionized (DI) water is mainly realized by a system that handles 18 MΩ cm (SHRO-plus DI). Calcium chloride dihydrate (CaCl_2_**·**2H2O) and ammonia bicarbonate (NH_4_HCO_3_) were purchased from Sinopharm Chemical Reagent CO, Ltd. phosphate buffer solution (PBS) were purchased from Thermo-Fisher (United States). Cell counting kit-8 (CCK-8), fluorescein diacetate (FDA), 1,1′-dioctadecyl-3,3,3′,3′-tetramethylindocarbocyanine perchlorate (Dil) and 4′,6-diamidino-2-phenylindole (DAPI) were purchased from Sigma-Aldrich in the United States. Others that need to be used are purchased from Aladdin reagent from China and Sinopharm Group.

**Synthesis of porous CaCO_3_ nanoparticles**

CaCO_3_ particles were synthesized by a gas diffusion reaction. Briefly, 150 mg CaCl_2_**·**2H_2_O was dissolved in 100 ml ethanol in a glass bottle covered by an aluminum foil which was punctured with several pores. Then, the bottle was put into a vacuum drying chamber containing 5 g dry ammonia bicarbonate (NH_4_HCO_3_). After keeping the whole system in a vacuum environment for 24 h, CaCO_3_ nanoparticles were obtained and could be separated by centrifugation at 8000 rpm. Those nanoparticles were re-dispersed in anhydrous ethanol for further modification. The prepartion of CaC consistent with the above experiments, just add 10mg Cur to the ingredients.

**Preparation of ECa and ECaC.**

The pure exosome were prepared according to the standard protocol of Exosome Isolation Reagent (RIBOBIO biotechnology co. LTD, China). For ECA preparation, CT26 cancer cells were cultured for 3 days and then the growth medium was rinsed out, replaced with fresh medium containing 1mg CaCO_3_ and incubated for 12 hours at 37 °C and 5% CO_2_ atmosphere. Then the medium was replaced by the fresh medium without FBS and the cells were incubated at 37 °C and 5% CO_2_ atmosphere for another 24 hours. Then all the supernatant was collected and centrifuged at 1125 g for 5 min to eliminate the cells and debris. The ECa in supernatant isolated by centrifuged at 10000 rpm for 5 min. ECa was stored at 4℃ for further use. The prepartion of ECaC consistent with the above experiments, CaCO_3_ NPs are replaced by CaC.

**Preparation of erythrocyte membranes biomimetic CaC nanoparticles (RCaC).**

The red blood cell-vesicles were obtained by using low-osmosis method. The RCaC were obtained by using an extrusion method. Briefly, 0.1 mg of CaC were mixed with 1 mg of red blood cell-vesicles, which were quantified by lyophilization and extruded through 100 nm polycarbonate membranes. The RCa were stored in PBS at 4 °C for the following experiments.

**Characterization of ECaC**

The morphologies of CaCO_3_ NPs and ECaC were analyzed by transmission electron microscopy (TEM, JEM-2010HT, Japan) under an accelerated voltage condition of 120 keV. High-angle annular dark-field scanning transmission electron microscopy (HAADF-STEM) images and corresponding energy-dispersive spectroscopy (EDS) mapping analyses were acquired with a field-emission TEM (JEM-F200, Japan). A UV/Vis-NIR spectrophotometer (UV-3600 in Shimadzu, Japan) was used for effective measurement of the light absorption spectrum. XRD was measured on a D8 Advance (Bruker- AXS, Germany). SDS-PAGE was also used to analyze the protein components onto nanoparticles.

**Degradation and drug release studies.**

To study the Ca^2+^ release, a solution of ECaC was dialyzed against PBS with PBS (pH 7.4), PBS (pH 6.5) or PBS (pH 5.5), respectively. The amounts of Ca2+ release at different time points were measured by ICP-AES.

**Cell culture and animal models**

CT26 colon cancer cell line was obtained from the Cell Bank of the Chinese Academy of Sciences and incubated in RPMI-1640 medium supplemented with 10% FBS in a humidified atmosphere at 37℃. Cell cultures under normoxic conditions (pO_2_: 21%) were maintained in a humidified incubator at 37℃ in 5% CO_2_ and 95% air.  CT26 (1 × 10^6^) in 100 μL of PBS in suspension was injected into all mouse bodies in the form of a subcutaneous injection of cells to complete the effective construction of tumor models. When the diameter of the tumor volume reached 200 mm^3^, the tumor-bearing mice were used for the next stage of the experiment. The animal experiments were strictly implemented based on the plan approved and released by the Ministry of Health of China and also approved by the Animal Research Management Committee of Wuhan University.

***In vitro* immune evasion study**

RAW 264.7 cells were seeded in 12-well plates and cultured for 12 h. Different concentrations (25, 50 and 100 μg/mL CaCO_3_) of RCaC, and ECaC were added the medium. Then the cells were incubated for 2 h at 37 °C, 5% CO_2_, and then washed with PBS three times. The nanoparticles uptake was measured by ICP-MS.

***In vitro* cancer targeting study**

CT26 cells were seeded in 24-well plates and cultured for 12 h. Different concentrations of RCaC and ECaC were added the medium. Then the cells were incubated at 37 °C, 5% CO_2_, and then washed with PBS three times. The cells were then fixed with PFA for 30 min at room temperature. The nanoparticles uptake was measured by ICP-MS as described above.

**Intracellular reactive oxygen species (ROS) generation**

For determination of ROS levels *via* fluorescent imaging, CT26 cells were incubated for 2 h with 5 different groups: (1) PBS (2) ultrasound (US, 1.0 MHz, 1.5 W/cm^2^, 50% duty cycle) (3) ECaC (4) US + ECa (5) US + ECaC. The CaCO_3_ concentration was 200 μg/mL in group 3, 4 and 5. Then, the fluorescent dye, DCFH-DA (10 μM), was added and co-incubated for 20 min at 37 °C. Then, cells in group 2, 4 and 5 were irradiated with the US. ROS level was determined by confocal laser scanning microscope (CLSM; IX81, Olympus, Japan). The fluorescent intensity of each group was calculated by ImageJ software.

**In vitro toxicity of CMC**

CT26 cells were seeded in 96-well plates at a density of 5 × 10^3^ cells per well and incubated for 24 h. Afterwards, CT26 cells were incubated for 2 h with 5 different groups: (1) PBS (2) ultrasound (US, 1.0 MHz, 1.5 W/cm^2^, 50% duty cycle) (3) ECaC (4) US + ECa (5) US + ECaC. The CaCO_3_ concentration was 200 μg/mL in group 3, 4 and 5. Then, cells in group 2, 4 and 5 were irradiated with the US. At the end of the incubation, 5 mg/mL MTT PBS solution was added, and the plate was incubated for another 4 h. Finally, the absorbance values of the cells were determined by using a microplate reader (Emax Precision, USA) at 570 nm. The background absorbance of the well plate was measured and subtracted. The cytotoxicity was calculated by dividing the optical density (OD) values of treated groups (T) by the OD values of the control (C) (T/C × 100%).

**In vivo pharmacokinetics**

BALB/c mice (n = 3) received an intravenous (i.v.) injection of 100 μL PBS containing CaC, RCaC or ECaC (with an equivalent Ca dose of 5 mg/kg). At various time points after the injection 20 μL blood plasma was collected from the tail veins and Ca^2+^ was quantitatively analyzed by ICP-MS.

**In vivo distribution study**

When tumors reached 200mm^3^, tumor bearing mice (n = 3) received an intravenous (i.v.) injection of 100 μL PBS containing CaC, RCaC or ECaC (with a Ca dose of 5 mg/kg). Mice were sacrificed at specific time points to collect the tumors and major organs for Ca2+ measurement by ICP-MS.

***In vivo* antitumor study**

When tumors reached 200mm^3^, tumor bearing mice were divided randomly into 5 groups (each group included 5 mice): (1) PBS (2) ultrasound (3) ECaC (4) US + ECa (5) US + ECaC. The CaCO_3_ concentration was 10 mg/kg in group 3, 4 and 5. The US was performed 12 h after intravenous injection. The treatment was conducted every 2 days for 14 days. Mice body weight was monitored every 4 days. After 16 days treatment, all the mice were sacrificed. Five main organs (heart, liver, spleen, lung and kidney) and tumors of all mice were harvested, washed with PBS, and fixed with paraformaldehyde for histology analysis. And the tumor tissues were imaged and weighed, and fixed in 4% neutral buffered formalin, processed routinely into paraffin, and sectioned at 4 μm. Then the sections were stained with Ki-67 and TUNEL and finally examined by using an optical microscope (BX51, Olympus, Japan).  Their blood samples and major organs (i.e., hearts, livers, spleens, lungs, and kidneys) were collected. Three important hepatic indicators (i.e., ALT: alanine aminotransferase, AST: aspartate aminotransferase, and ALP: alkaline phosphatase) and two indicators for kidney functions (i.e., BUN: blood urea nitrogen and CRE: creatinine) were measured by using a blood biochemical autoanalyzer (7080, HITACHI, Japan).

**Statistical analysis**

Data analyses were conducted using the GraphPad Prism 5.0 software. Significance between every two groups was calculated by the Student’s t-test. *P < 0.05, **P < 0.01, ***P < 0.005.


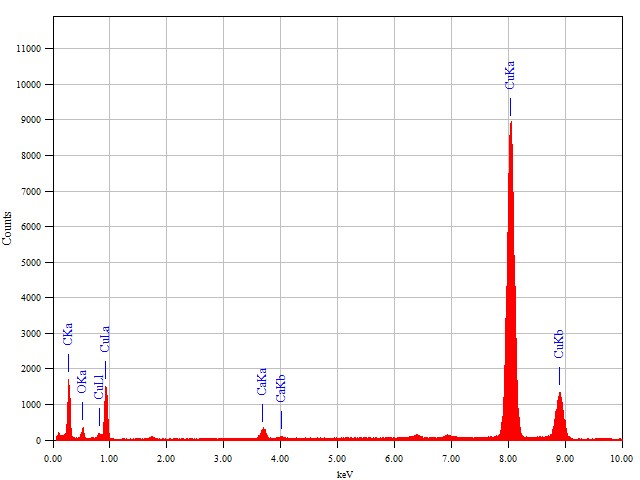


**Figure S1.** EDX of CaCO_3_ NPs.


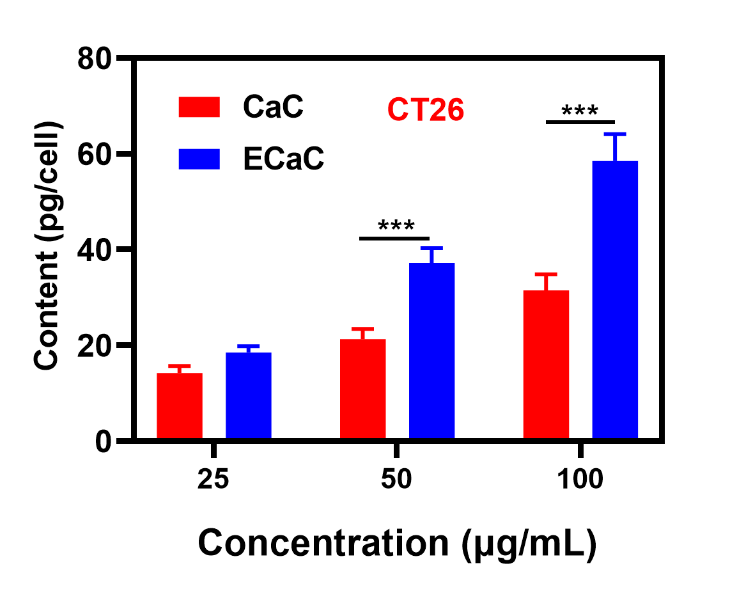


**Figure S2.** Nanoparticle uptake by CT26 cells at different incubated concentration (CaCO_3_ dose of 25, 50, and 100 μg/mL).


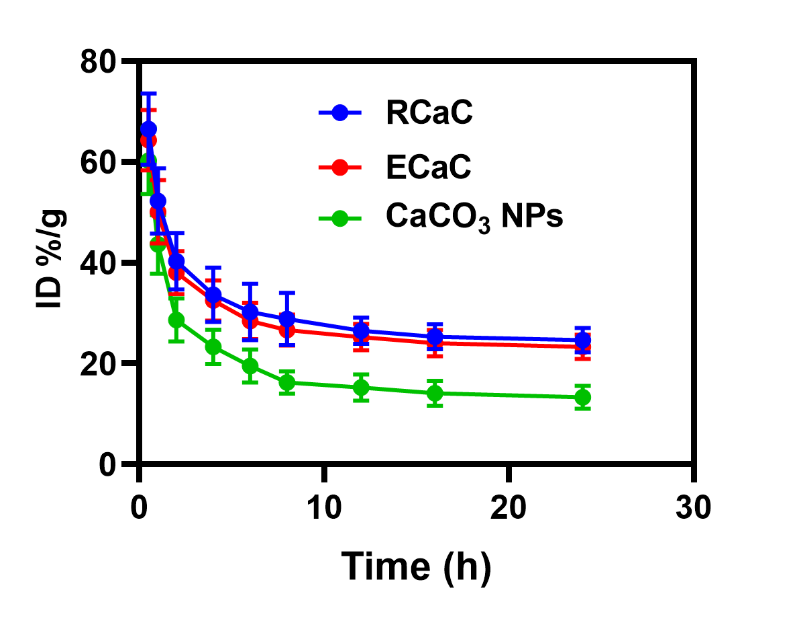


**Figure S3.** Pharmacokinetic behavior of CaCO3, RCaC, and ECaC in mice following i.v. administration at doses of 5 mg Ca/kg.


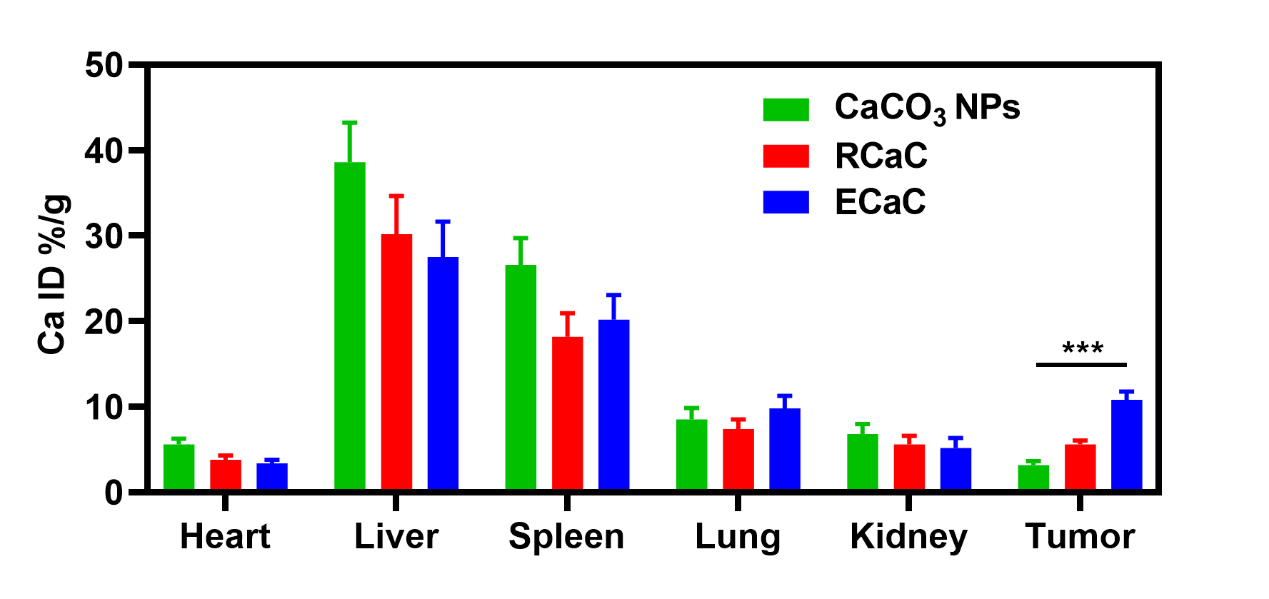


**Figure S4.** Quantitative analysis of Ca biodistribution in tissues and tumors of tumor-bearing mice injected with CaCO3, RCaC, and ECaC at CaCO3 dose of 5 mg Ca/kg, respectively.


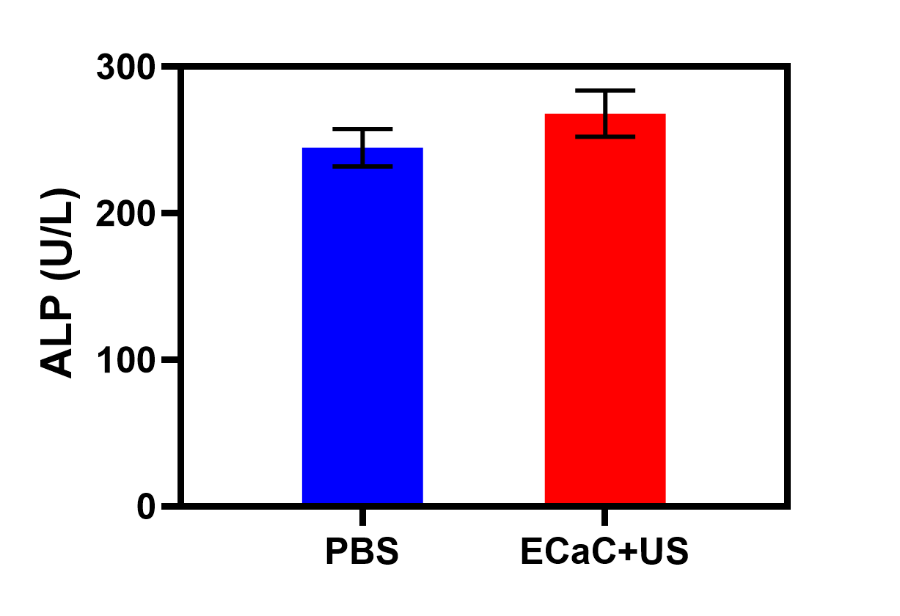


**Figure S5.** Blood biochemistry data of kidney and liver function markers: ALP.


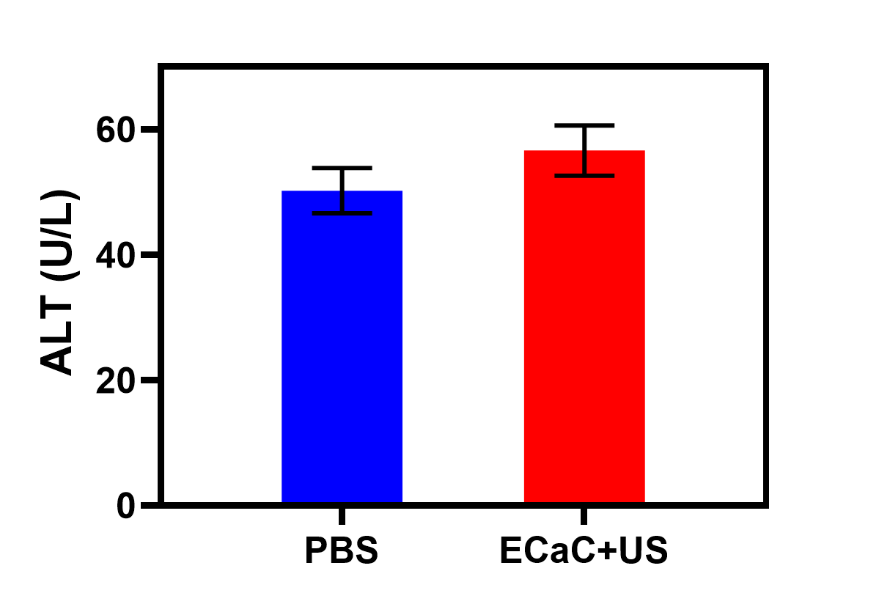


**Figure S6.** Blood biochemistry data of kidney and liver function markers: ALT.
